# Presence of atypical genotypes of *Toxoplasma gondii* isolated from cats in the state of Bahia, Northeast of Brazil

**DOI:** 10.1371/journal.pone.0253630

**Published:** 2021-10-05

**Authors:** Ana Graziela de Jesus Deiró, Danielle Paschoal do Prado, Iuri Prates Sousa, Daniele de Santana Rocha, Rodrigo Alves Bezerra, Fernanda Amato Gaiotto, George Rego Albuquerque, Alexandre Dias Munhoz

**Affiliations:** 1 Graduation Program in Animal Science, Santa Cruz State University, Ilhéus (BA), Brazil; 2 Graduate Student in Veterinary Medicine, Santa Cruz State University, Ilhéus (BA), Brazil; 3 Department of Agricultural and Environmental Sciences, Santa Cruz State University, Ilhéus (BA), Brazil; 4 Ilheus College, Ilhéus (BA), Brazil; 5 Molecular Markers Laboratory, Center for Biotechnology and Genetics, Santa Cruz State University, Ilhéus (BA), Brazil; Institut national de la santé et de la recherche médicale - Institut Cochin, FRANCE

## Abstract

In this study, 20 blood, heart, and brain samples were collected from euthanized cats at the Zoonosis Control Centers and Veterinary Clinics in the state of Bahia, Brazil. The sera were examined for anti-*T*. *gondii* antibodies using the indirect hemagglutination test. The brains and hearts of seven seropositive cats were ground, and peptide digestion was performed for bioassay in mice. *Toxoplasma gondii* was isolated in 5/7 (71.42%) of seropositive cats. In these isolates, the parasite was genotyped using the Polymerase chain reaction, associated with the DNA fragment polymorphism obtained by restriction enzyme PCR-RFLP technique with 11 markers (SAG1, 5’-SAG2, 3’-SAG2, alt. SAG2, SAG3, BTUB, GRA6, c22-8, c29-2, L358, PK1, Apico, and CS3) and 15 microsatellite markers (TUB-2, W35, TgM-A, B18, B17, M33, IV.1, XI.1, M48, M102, N60, N82, AA, N61, N83). The analysis of the isolates by PCR-RFLP revealed five distinct genotypes. Three of these genotypes have never been reported before; one corresponded to the TgDgCo13 genotype, and one incomplete genotype. In genotyping analysis using microsatellite markers, it was observed that the isolates showed atypical alleles in the typing and fingerprint markers. This revealed five atypical genotypes. The typing marker B17 showed the highest degree of atypia. This study is the first to report the genotyping of *T*. *gondii* obtained from naturally infected cats in Bahia, Northeast Brazil. The genotypes found in this study were different from those found in other studies conducted in Bahia, which included different species of animals. None of the clonal lineages I, II, or III were found. This study demonstrates the diversity of *T*. *gondii* in the study region, with the presence of unusual genotypes, reaffirming the genetic variability of the parasite in Brazil.

## Introduction

*Toxoplasma gondii* is a coccidium that has a cosmopolitan distribution and can affect humans and other homeothermic species. It has great relevance in public health because it causes toxoplasmosis. Toxoplasmosis is a zoonosis that causes eye changes, congenital diseases, and serious complications in humans and animals that can lead to death [1, 2]; felines are the definitive hosts for *T*. *gondii* [3, 4].

The parasite has a high genetic diversity; studies have established the grouping of this parasite into three genotypes, Ι, ΙΙ, and ΙΙΙ in North America and Europe [5]. Genotype Ι consists of highly virulent strains for all hosts; these strains are the most isolated in cases of congenital toxoplasmosis in humans. Genotypes II and III, which are less virulent, are cystogenic [6].

In Central and South America, studies on domestic and wild animals have shown that the vast majority of strains are unique [7]. In Brazil, samples belonging to genotypes I, II, and III have been identified. However, this region is characterized by the presence of other combinations referred to as atypical genotypes. These tend to result in more serious infections than those in countries from North America, Europe, and Africa. These data were obtained from isolates from animals, including cats, using the polymerase chain reaction, associated with the DNA fragment polymorphism obtained by restriction enzyme (PCR-RFLP) technique and the typing of microsatellite markers [8–12]. The use of these techniques demonstrated that the isolates from Brazil do not fit into an exclusively clonal propagation pattern, showing a high genetic diversity [10, 13, 14].

Because most of the *T*. *gondii* cycle is clonal (haploid), and a small diploid passage occurs in the definitive hosts (Dubey, 2008), the study of felines becomes important either in the clonal dispersion or in the genetic recombination of the parasite in the intestine. This is especially true in regions where atypical and recombinant strains are common.

This study aimed to isolate *T*. *gondii* from cats from the mesoregions of Salvador and Sul Baiano, Northeast Brazil. In addition, the aim of the study was to genetically characterize the isolates using PCR-RFLP, along with the typing of 11 markers and 15 microsatellite markers to compare the strains found herein with those isolated in previous studies in the same region to verify their diversity.

## Materials and methods

### Sample collection and serological testing

This project was approved by the Animal Use Ethics Committee protocol 04/2018 of the State University of Santa Cruz (UESC). Twenty cats (from Zoonosis Control Centers or localized vetirinary clinics in the mesoregion of Salvador and south Baiano state of Bahia, Brazil), that had a pre-existing disease, were euthanized in order to harvest the blood, brain, and heart and the samples were properly packed, during April to December 2018.

Blood samples were collected in tubes without anticoagulant. They were then centrifuged at 1,465 *g* for 10 min at 25°C to obtain the serum which was used for the detection of antibodies against *T*. *gondii* by the hemagglutination test (Immuno-HAI Toxoplasmosis Wana Diagnóstica®) with a cutoff point of 1:64 [15]. The heart and brain samples of each cat were individually placed in clean collectors containing saline solution (0.9% NaCl) and an antibiotic solution (1,000 IU penicillin G potassium and 100 mg streptomycin) and were refrigerated until biological tests were carried out.

### Isolation of *Toxoplasma gondii*

The brains and hearts of each seropositive cat were homogenized and subjected to peptic digestion [16–18]. A 1-mL aliquot of the digestion product was inoculated intraperitoneally and subcutaneously into albino mice. Four mice were inoculated by a seropositive cat (2 intraperitoneal and 2 subcutaneous). The mice were fed a conventional diet and were given water *ad libitum* and observed daily for 60 days. Those who showed a change in behavior (such as apathy, lethargy, bristly hair, or increased abdominal volume) were immediately euthanized using the cervical dislocation technique and examinations were performed to visualize the parasite in peritoneal fluid, and to print organs on a histological slide. When *T*. *gondii* tachyzoites were observed in the liquid, they were frozen in DMSO and fetal bovine serum for subsequent DNA extraction.

Mice that did not become ill were euthanized 60 days after inoculation using the cervical dislocation technique. This was carried out by a veterinary medical professional with necessary qualifications and the ability to execute the mentioned technique with due care to occupational health and safety. The brains of these animals were imprinted to view the cysts. Samples of the organs (brain, heart, lung, liver, and spleen) were collected from all mice for subsequent DNA extraction.

### DNA extraction from tissue samples of inoculated mice

Fragments of 100 mg of the spleen, liver, heart, lung, and brain of all mice were macerated individually, and DNA was extracted with an Easy-DNA kit® (Invitrogen, Carlsbad, California, USA) with protocol number 3 of kit.

### Polymerase Chain Reaction (PCR) for detection of *Toxoplasma gondii*

Amplification of *T*. *gondii* DNA was performed using the method described by Homan et al. (2000). Primers Tox4 (CGCTGCAGGGAGGAAGACGAAAGTTG) and Tox5 (CGCTGCAGACACAGTGCATCTGGATT) were used resulting in a 529-bp fragment (GenBank No. AFI46527) of *T*. *gondii* DNA [17–19]. A PCR reaction was performed in 25 μL of total volume containing 1x PCR buffer, 2 mM MgCl_2_, 200 μM dNTPs, 0.30 μM of each primer (forward and reverse), 0.5 U of Taq DNA Polymerase Platinum®, 1.5 μL of extracted DNA, and ultrapure Milli-Q water to complete the volume. Thermocycling conditions were as follows: 95°C for 4 min, followed by 35 cycles at 94°C for 30 s, 60°C for 1 min, and 72°C for 1.5 min. This was followed by a final extension at 72°C for 2 min. The PCR products were analyzed by electrophoresis on a 2% agarose gel which was stained with SYBR® Safe (Invitrogen) and photo-documented by LPix Image-Loccus Biotechnology. RH tachyzoites were used as the positive controls. The negative control consisted of DNA extracted from brain samples from uninoculated mice.

### Genotyping of *Toxoplasma gondii* using the PCR-RFLP technique

Genotyping of *T*. *gondii* was carried out using the PCR-RFLP technique [18–20]. A few modifications were made wherein the ringing temperature of the nested PCR (nPCR) ranged from 58°C to 60°C. A primary PCR reaction was performed in total volume of 25 μL containing 2.5 μL PCR buffer (10x), 1.0 μL MgCl_2_ (50 mM), 200 μM dNTPs, 0.10 μM of each primer (direct and reverse), 0.5 U of Taq DNA Polymerase Platinum®, 1.5 μL of extracted DNA and ultrapure water (Milli-Q) to complete the volume. The reaction was incubated at 95°C for 4 min followed by 25 cycles at 94°C for 30 s, 55°C for 30 s, and 72°C for 1.5 min. This was followed by a final extension at 72°C for 2 min.

The PCR products were used for nPCR. Each 25 μL reaction contained 2,5 μL of PCR buffer (10x), 1,0 μL MgCl_2_ (50 mM), 200 μM of each dNTP, 0.3 μM of each primer used separately (direct and reverse), 0.5 U of Taq DNA Polymerase Platinum®, 1.5 μL of product PCR and ultrapure Milli Q^®^ water to achieve the final volume. The reactions were carried out under the following conditions: 95°C for 4 min followed by 35 cycles at 94°C for 30 s, 60°C for 1 min, and 72°C for 2 min. This was followed by a final extension at 72°C for 2 min.

The nPCR products were treated with their respective restriction enzymes, analyzed by electrophoresis in 2%, 2.5%, and 3% agarose gels and was stained with SYBR® Safe (Invitrogen). A photo was taken to document the PCR-RFLP standards of the isolates (S1 Table). Each isolate was characterized using genetic markers: SAG1, 5’-SAG2, 3’-SAG2, alt. SAG2, SAG3, BTUB, GRA6, c22-8, c29-2, L358, PK1, Apico, and CS3 [13].

Two negative controls were used: a strain of human foreskin fibroblasts (HFF) and ultrapure Milli-Q^®^ water. The positive controls used included: reference strains of type I (RH), type II (PTG), type III (CTG), and atypical strains (TgCgCa1, MAS, and TgCatBr5). The DNA band patterns of the samples were compared to those of the genotypes deposited in ToxoDB (http://toxodb.org/toxo/). Isolates that did not show bands against any genetic markers were considered to be incomplete.

### Analysis of clusters and diversity of *Toxoplasma gondii* isolates

The dendrogram was built based on the band patterns obtained through PCR-RFLP using the 11 genetic markers tested using the nearest neighbor clustering algorithm with the aid of the PAST program version 2.17c.

The cluster analysis was based on a binary matrix that represented the presence/absence of bands in a sample. The similarity matrix was obtained using the similarity coefficient (SD) data. SD (values between 0 and 1) is an arithmetic determination of the degree to which the band patterns are similar (i.e., they contain the same bands). The SD was calculated using the formula SD = 2nAB / (nA + nB), where nA is the number of PCR-RFLP bands in line A, nB is the number of bands in line B, and nAB is the number of bands common to lines A and B. The groupings were determined by sequential comparison of the patterns and construction of a dendrogram reflecting the relative similarities using the Dice method.

Using the K-means cluster tool, it is possible to group the found genotypes, dividing clusters, and demonstrating the differences and similarities of the genotypes.

The isolates of *T*. *gondii* from cats were analyzed, and the strains of *T*. *gondii* were used as controls in this study (RH, PTG, CTG, TgCgCa1, MAS, and TgCatBr5).

### Genotyping of *Toxoplasma gondii* by microsatellite markers

For the analysis of microsatellites of isolated strains, conventional PCR was performed using 15 microsatellite markers (TUB-2, W35, TgM-A, B18, B17, M33, IV.1, XI.1, M48, M102, N60, N82, AA, N61, N83) from Applied Biosystems which are located on 11 different chromosomes from *T*. *gondii* [6] (S2 Table). A mix containing 1 μL DNA (extracted from mouse ascitic fluid, from mouse brain bradyzoites and other mouse and animal tissues) was prepared for each PCR reaction, 0.2 μL of each dNTP (10 mM), 0.5 μL MgCl_2_ (50 mM), 1.0 μL buffer (10x), 0.2 μL of Taq Platinum DNA polymerase, 5 mM of the reverse primer, 5 mM of the forward primer and ultra-pure water at a final volume of 12 μL. The amplification reaction was performed in a Veriti® 96-Well Thermal Cycler (Applied Biosystems) under the following conditions: 15 min of initial denaturation at 95°C, followed by 35 cycles of 30 s at 94°C, 3 min at 61°C, 30 s at 72°C and a final extension of 30 min at 60°C. The amplification reaction was verified using a 2% agarose gel. After standardization, the amplicon was used for genotyping in an automatic sequencer (ABI PRISM 3500; Applied Biosystems). Three reference strains were used: ENT (type I, France), ME49 (type II, USA), NED (type III, France), the archetypal reference strains of humans, goats, and humans, respectively.

### Genotyping in the automatic sequencer

Two markers were combined in each race, which were analyzed simultaneously to reduce costs and time. For panel assembly, each well of the genotyping plate received 2 μL of the PCR product mixture from two amplified loci, plus 7.8 μL of formamide and 0.2 μL of the standard size marker LIZ-500 (Applied Biosystems). The plate was denatured in a Veriti® 96-Well Thermal Cycler (Applied Biosystems) at 94°C for 3 min and then placed on ice for 5 min for thermal shock. Capillary electrophoresis was performed using an ABI 3500 Genetic Analyzer (Applied Biosystems). Subsequent analyses for determining allele sizes (genotyping) were performed using GeneMarker software (version 2.6.0; Soft Genetics, State College, PA, USA).

### Phylogenetic analysis

The tree was built from microsatellite data. In order to carry out a comparison, in addition to the samples from this study, we included data on genotyping of strains previously analyzed in other studies carried out in Brazil and in the world (S4 Table). The strains ENT, ME49 and NED were used as reference for strains of T. gondii type I, II and III, respectively. Populations 1.2.32 (http://bioinformatics.org/populations/) was used based on the Dc chord-distance evaluation by Cavalli-Sforza and Edwards and generated with MEGA version 6.05 (http://www.megasoftware.net/history.php).

## Results and discussion

Seven (35%) of the twenty cats had anti-*T*. *gondii* antibodies. Therefore, 28 mice were required to perform the experiment. Isolation was possible through bioassays in five cats (Table 1). The performance of PCR-RFLP with 11 genetic markers revealed four distinct genotypes (TgCatBr85, 86, 88, 89), one incomplete (TgCatBr87), and no band formation against two markers (GRA6 and PK1). Two isolates (TgCatBr86 and 88) were virulent in mice, with the appearance of clinical signs between 7 and 14 days after inoculation. The two other isolates (TgCatBr 85 and 87) were the only ones that caused encystment. The behavior of TgCatBr89 was different from that of the other isolates. It caused the disease in mice that were inoculated intraperitoneally and caused encystment in one of the two mice that were inoculated subcutaneously.

**Table 1:** Detection of *Toxoplasma gondii* by PCR in samples from naturally infected cats, associated with the bioassay.

Isolated					PCR mice
		Clinical signs in mice			

	Day of euthanasia	Mice IP*	Mice SC**	Cyst presence / Total	Positive / Total
**TgCatBr85**	d60	0/2	0/2	4/4	4/4
**TgCatBr86**	d7-d14	2/2	2/2	0/4	3/4
**TgCatBr87**	d60	0/2	0/2	4/4	4/4
**TgCatBr88**	d7-d14	2/2	2/2	0/4	4/4
**TgCatBr89**	d7-d14/ d60***	2/2	1/2	1***/4	3/4

*Mice IP–mice inoculated intraperitoneally

**Mice SC—mice inoculated subcutaneously

*** encysting in one of the two mice inoculated subcutaneously

Strains isolated from the southern Bahia region were considered virulent, compared to those from the Salvador region. The TgCatBr 86 and 88 isolates found in this study were grouped in cluster I. The isolates TgCatBr 85, 87, and 89 were grouped in cluster III (Table 2).

**Table 2:** Grouping of the genotypes found, showing similarities with the already known genotypes, using K-means cluster.

Isolated	Cluster
**TgCatBr85**	III
**TgCatBr86**	I
**TgCatBr87**	III
**TgCatBr88**	I
**TgCatBr89**	III
**RH**	I
**PTG**	II
**CTG**	III
**TgCgCa1 COUGAR**	II
**MAS**	I
**TgCatBR5**	I

Through cluster analysis, the isolates were grouped into two clusters, Type I and Type III, based on the similarities of the isolates within each type.

**Fig 1:**
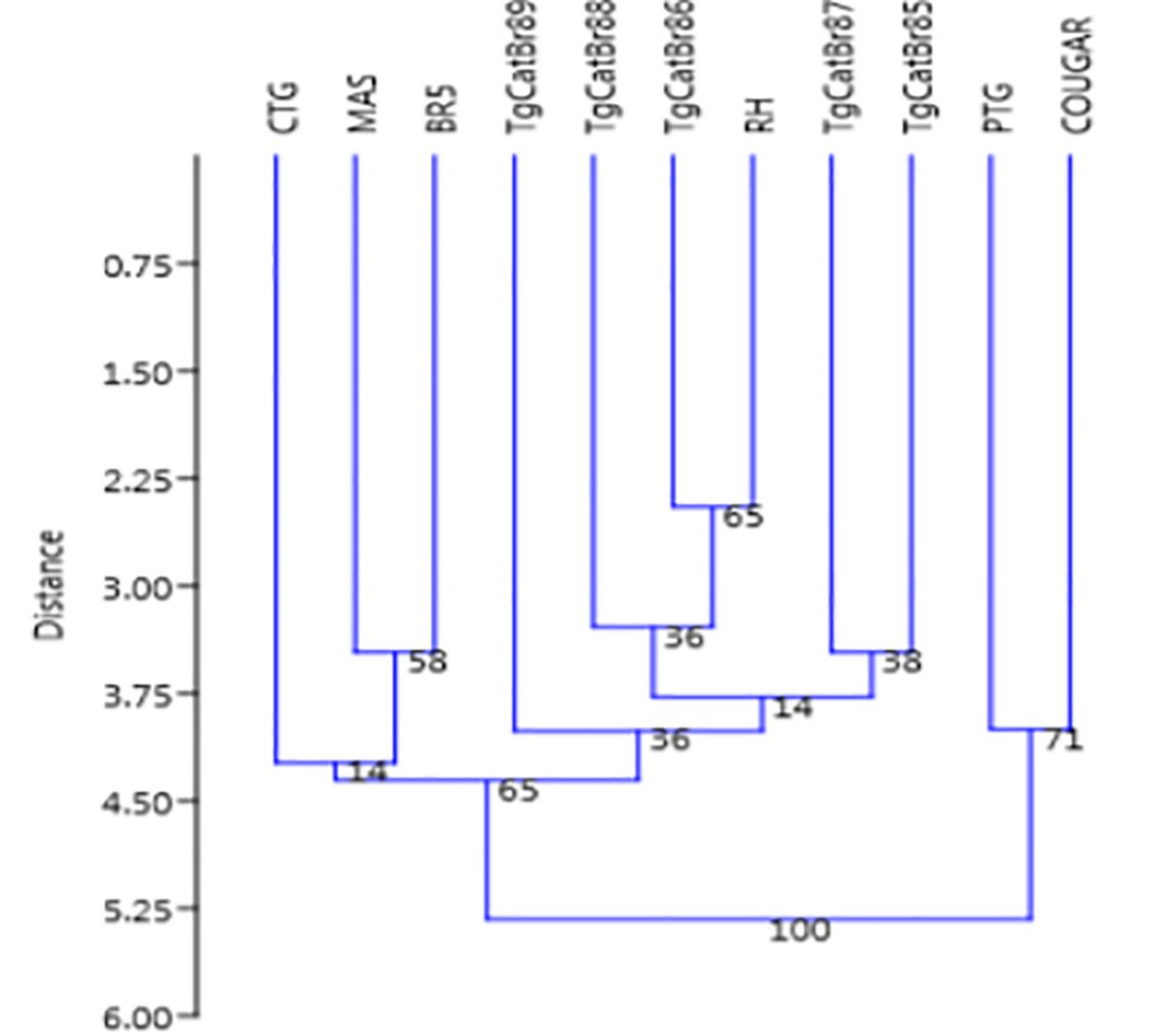
Dendrogram of *Toxoplasma gondii* strains constructed based on the PCR-RFLP band patterns using eleven genetic markers (SAG1, 5’-SAG2, 3’-SAG2, alt. SAG2, SAG3, BTUB, GRA6, c22-8, C29-2, L358, PK1, Apico and CS3). The tree was built using the neighbor-joining method for clustering after bootstrapping with 10,000 repetitions, and the distances were calculated using the Dice method.

The dendrogram of the genotypes, as assessed by the neighbor-joining method, demonstrated that the isolates in this study were close to the RH isolates (closest to Type I) (Fig 1).

In the analysis of the virulence marker CS3, four isolates in which this marker was specific for Type I were from Mesorregião Sul Baiano and a single isolate that presented allele u-1 belonged to Mesorregião de Salvador. The TgCatBr87 isolate, in which this marker was specific for Type I, caused no illness in the mice, thereby demonstrating a behavior that was different from that of the other isolates.

The molecular analysis of 11 markers in the five isolates by PCR-RFLP showed the presence of three unique genotypes that were different from those found in our studies. This suggested a high genetic diversity in the population of circulating *T*. *gondii*, corroborating the results of the genotyping of isolates from other animals in the same state [21–25] and in other states of Brazil [12–14] (S3 Table). Only one genotype (TgCatBr 85) was previously described by a single isolate found in a dog in Colombia by Chanlei Su in 2016. This diversity is the result of the high rate of the sexual recombination of these parasites [2, 13].

The isolates from Brazil do not fit into an exclusively clonal propagation pattern; this indicates a frequent incidence of genomic recombination that generates diversity [6, 13, 14]. These isolates may have new mechanisms of pathogenicity, resulting in a new genotype that exhibits greater pathogenicity and causes more severe cases of toxoplasmosis in immunocompetent individuals. In addition, it causes emerging diseases with its expansion.

The circulation of many genotypes in Brazil is justified by the high degree of biodiversity in this region, the large number of feline species found in Brazil, the large number of stray cats, and the close contact among animals infected with *T*. *gondii* [3], allowing recombination.

The virulence of these isolates was assessed through parasite-mouse interactions, and this relationship can be correlated with the genotype of the isolate [21, 24, 25]. Three isolates from this study were virulent in mice, similar to the results of other studies [24, 25]. These isolates showed allele I at the CS3 locus. The virulence of these isolates observed in this study may be related to the fact that these isolates have type I alleles in several markers. These findings are consistent with the illness observed in infected mice. The type I allele is often associated with high and intermediate pathogenicity, causing clinical changes in mice, unlike allele III, which is responsible for the survival of these animals [13, 14, 26].

The CS3 marker can be widely used as an indicator of *T*. *gondii* virulence as analyzing it is a potentially faster and more practical alternative to performing the biological assay in mice [13, 24–26]. The results of the cluster analysis corroborated the findings of other studies in Brazil that demonstrated that there were few atypical or recombinant isolates associated with genotype II [12–15, 21, 22, 25].

The presence of atypical alleles can affect the phenotype of recombinant strains, resulting in greater virulence. A recombinant may have new pathogenic mechanisms, resulting in a new genotype that exhibits greater pathogenicity in mice and more severe cases of toxoplasmosis in immunocompetent individuals. This may also cause emergent diseases with its expansion [27].

In genotyping analysis using microsatellite markers (MS), it was observed that the isolates showed atypical alleles in the typing and fingerprint markers (S4 Table). The presence of atypical alleles was observed in the typing markers in all the analyzed isolates, and the marker B17 showed the highest degree of atypia. As already described in studies in Brazil, the fingerprint markers demonstrated that all the analyzed genotypes were distinct [14, 15, 28].

MS behaved as expected with regard to the distinction between clonal types and in the identification of atypical alleles. We observed the presence of atypical alleles in the typing (TUB2, B18, and B17) and fingerprint markers. This further corroborated the high diversity of *T*. *gondii* strains from Brazil and South America where the isolates have atypical characteristics [6, 11, 12, 17, 21]. Moreover, the studies by Can et al. (2014) in Turkey (Eurasia) reported isolates type II, type III, and genotype Africa 1, which corroborates the results of other studies carried out in this region [6].

Valenzuela-Moreno and his collaborators (2020), in their study in Mexico, found two isolates in dogs. These showed atypia in a typing marker (IV-1) and were considered new genotypes. This is in agreement with other studies carried out in this region [6].

In the fingerprint markers, we observed the presence of atypical alleles, mainly in the markers M48, M102, N60, and AA. Silva et al. (2017) genotyped strains of *T*. *gondii* in cats, and three rats on the Fernando de Noronha Island- Brasil. Within each group, differences between the strains were limited. Only TgMA (typing microsatellite marker) was able to differentiate the strains with the ToxoDB-RFLP # 146 genotype in the type III group. The microsatellite fingerprint markers M48 and N61 differentiated two isolates with genotype ToxoDB-RFLP n° 3 belonging to the type II group. In addition, the allele 329 was seen in the microsatellite marker N83; this allele is absent in strains in Europe and North America and has thus far been identified only in Brazilian strains. One isolate with genotype ToxoDB-RFLP n° 13 belonged to the Caribe1 group.

**Fig 2:**
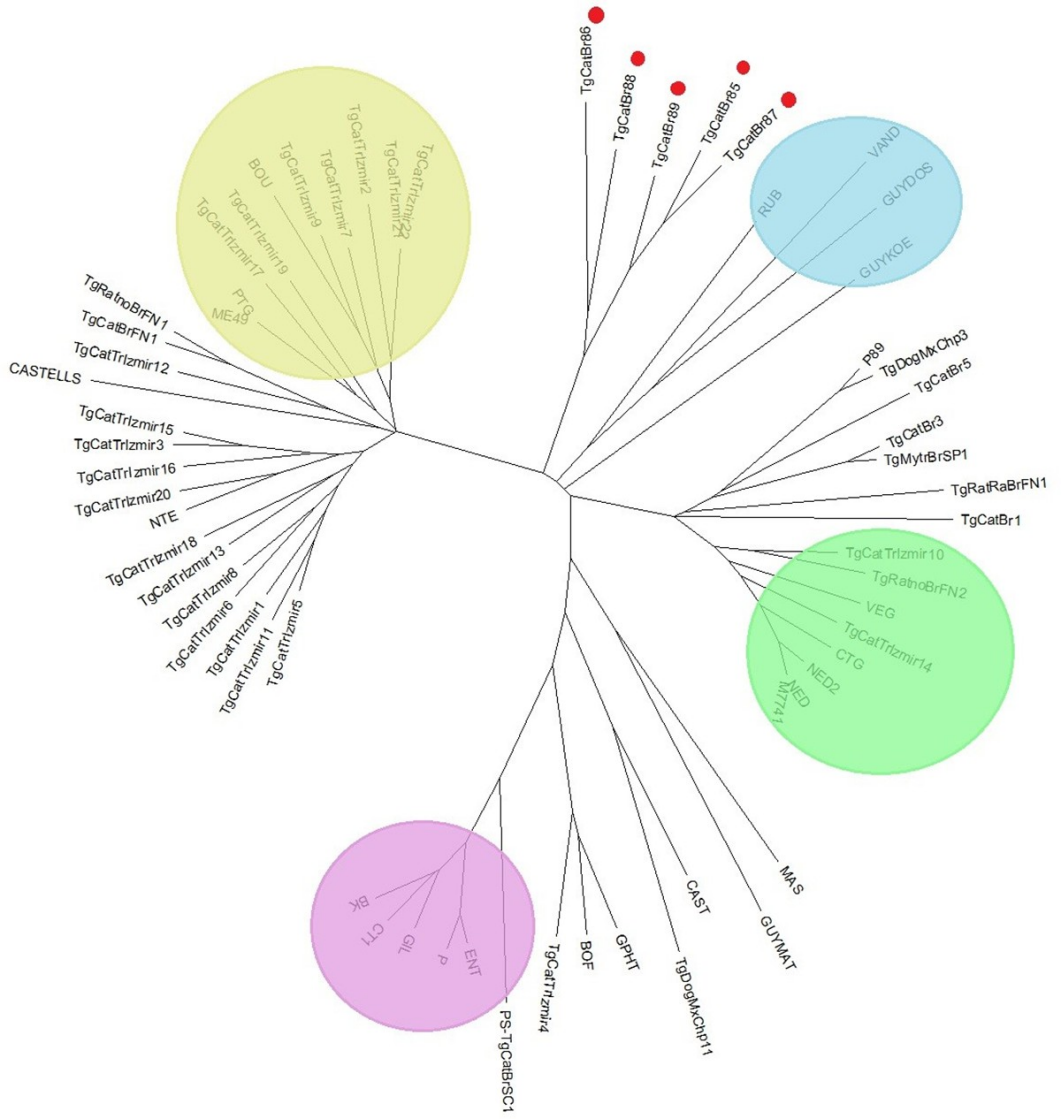
Neighboring cluster of Toxoplasma gondii strains based on 15 microsatellite markers. The lilac color indicates the type I (ENT), beige type II (ME49) and green type III (NED), which are the reference strains; The blue color indicates atypical strains found in French Guiana. The red dots indicate strains from the present study.

The samples analyzed in this study are atypical and differ from the samples previously studied in Brazil and worldwide. Analyzing the genetic distance between the individuals analyzed with samples from other studies, there is a greater proximity to the atypical individuals located in French Guiana (GUY-KOE, VAND, GUY-DOS, RUB), being different and genetically distant from type I, II and III, and thus presenting a greater amount of atypia. It is possible to observe an approximation of the samples analyzed with type II and III, this approximation being greater with type III (Fig 2).

The typing marker B17 proved to be a good marker for atypia compared to the other typing markers. Biologically, it is a good atypical allele marker as found in the work of Ajzemberg et al., 2010 and Pena et al., 2020.

The high genetic diversity of *T*. *gondii* in South America and Brazil may be due to random crosses, with greater opportunities for the genetic recombination of this agent in domestic and wild cats. This may explain the existence of a significant number of strains of this parasite. Moreover, it has been present on this continent for a very long time. This evolutionary history may have allowed the constitution of a substantial gene pool through mutations and sexual recombination [29]. We assume that the atypical genotypes found in cats in this study may also be circulating in humans and other animals because they live close by and can share sources of infection. This can lead to severe toxoplasmosis. New studies should be conducted for a better understanding of the issues of gene exchange and recombination.

We believe that felids that inhabit regions with a high presence of recombinant and atypical strains can excrete oocysts more than once in their lifetime. Cats reinfected with different clonal strains can excrete oocysts in small amounts again due to potentially incomplete cross-immunity [29]. This would generate a vicious cycle, with environmental contamination by different clones or their recombination; further, an intermediate host infected by these strains can be preyed upon by another felid, thereby resulting in a new recombination in the intestine of the felid.

Studies in some regions of Brazil, Colombia, and northern Argentina have reported a high prevalence of severe forms of toxoplasmosis affecting the human eye; this represents a significant public health problem [30–32]. These serious forms of ocular toxoplasmosis are related to pathogenic strains of *T*. *gondii* circulating in South America in association with the genetic diversity of this parasite on this continent [33, 34].

The current globalization and maritime trade, with the transport of contaminated meat products, domestic animals or other animals infected with *T*. *gondii*, in addition to migratory birds, can contribute to the intercontinental spread of new strains of this parasite. This can potentially direct the flow of this spread from South America to other continents and vice versa. In addition, this can also cause major public health challenges.

## Conclusion

We observed high genetic variability of *T*. *gondii* in the analyzed samples, corroborating with studies on parasite diversity in Brazil and in South America, where clonal genotypes are common. In this study, the marker CS3 was useful for evaluating the virulence of this parasite in mice. The microsatellite marker B17 showed atypia in four of the five isolates, suggesting that it may serve as a good marker of atypical alleles. Through analysis using two genotyping techniques, it was proven that the *T*. *gondii* isolates from cats in municipalities in Bahia were atypical.

## Supporting information

S1 Table Summary of markers, gene, location and enzymes used in PCR-RFLP (Restriction Fragment Length Polymorphism) (Su et al., 2010). * CutSmart Buffer® was used for all reactions; **F: forward *primer*; ***R: reverse *primer*.
(PDF)

S2 Table Microsatellite markers and PCR primers used for the PCR assay. a) Based on the position within each chromosome of the ME49 strain, as indicated on the Toxo DB website (http://toxodb.org). b) (F), direct initiator; (R), reverse primer. In each primer, the direct primer was labeled in part 5 with fluoroscein: 6-carboxyfluorescein (6-FAM) for MS TUB2, XI.1, B18, N83, N61, M33 and M48, hexachlorofluorescein (HEX) for MS TgM -A, B17, N82, W35 and IV.1 and 2,7,8-benzo -5-fluoro-2, 4,7-trichloro-5-carboxyfluorescein (NED) for MS AA, N60 and M102. SOURCE: Ajzemberg et al., (2010).
(PDF)

S3 Table Different *T*. *gondii* genotypes using PCR-RFLP in Bahia, Brazil. u-1, u-2 –atypical alleles; Nd -not determined.
(PDF)

S4 Table Genotyping by MS of Toxoplasma gondii isolates from cats in Bahia-Brazil and compared to isolates from other studies. Three archetypal reference strains (ENT, ME49 and NED) are included. * bold atypical alleles.
(PDF)
