# Supplementary material for: Presence of atypical genotypes of Toxoplasma gondii isolated from cats in the state of Bahia, Northeast of Brazil
Source: PLoS One. 2021 Oct 5;16(10):e0253630. doi: 10.1371/journal.pone.0253630 (PMC8491887; doi:10.1371/journal.pone.0253630)
Supplement: S1 Table — * CutSmart Buffer® was used for all reactions; **F: forward primer; ***R: reverse primer. (PDF) [file pone.0253630.s001.pdf]

S1 Table. Summary of Markers, gene, location and enzymes used in PCR-RFLP (Restriction Fragment Length Polymorphism) (Su et al., 2010)

Marker	Gene	Location	Multiplex PCR *primers*	nPCR *primers*	nPCR (bp)	Restriction enzymes	Incubation time and temperature	Reference
**SAG1**	SAG1	chromosome VIII	F**:GTTCTAACCACGCACCCTGAG<br>R***:AAGAGTGGGAGGCTCTGTGA	F: CAATGTGCACCTGTAGGAAGC<br>R:GTGGTTCTCCGTCGGTGTGAG	390	Sau96I + HaeII	37°C 1h. 2,5% gel	Grigg et al., 2001b ; Khan et al., 2005a
**5'SAG2**	SAG2	chromosome VIII	It is not necessary. The DNA fragment for 5'SAG2 is covered by the external alt.SAG2 primers.	F:GAAATGTTTCAGGTTGCTGC<br>R:GCAAGAGCGAACTTGAACAC	242	MboI	37°C 1h. 2,5% gel	Howe et al., 1997; Khan et al., 2005a
**3'SAG2**	SAG2	chromosome VIII	F:TCTGTTCTCCGAAGTGACTCC<br>R:TCAAAGCGTGCATTATCGC	F:ATTCTCATGCCTCCGCTTC<br>R:AACGTTTCACGAAGGCACAC	222	HhaI	37°C 1h. 2,5% gel	Lehmann et al., 2006 Khan et al., 2005a
**alt.SAG2**	SAG2	chromosome VIII	F:GGAACGCGAACAATGAGTTT<br>R:GCACTGTTGTCCAGGGTTTT	F:ACCCATCTGCGAAGAAAACG<br>R:ATTTCGACCAGCGGGAGCAC	546	HinfI +TaqI	37°C 30min, 65°C 30 min. 2,5% gel	Grigg et al., 2001b; Khan et al., 2005a
**SAG3**	SAG3	chromosome XII	F:CAACTCTCACCATTCCACCC<br>R:GCGCGTTGTTAGACAAGACA	F:TCTTGTCGGGTGTTCACTCA<br>R:CACAAGGAGACCGAGAAGGA	225	NciI	37°C 1h. 2,5% gel	Khan et al., 2005a
**BTUB**	BTUB	chromosome IX	F:TCCAAAATGAGAGAAATCGT<br>R:AAATTGAAATGACGGAAGAA	F:GAGGTCATCTCGGACGAACA<br>R:TTGTAGGAACACCCGGACGC	411	BsiEI + TaqI	60°C 1h. 2,5% gel	Khan et al., 2005a
**GRA6**	GRA6	chromosome X	F:ATTTGTGTTTCCGAGCAGGT<br>R:GCACCTTCGCTTGTGGTT	F:TTTCCGAGCAGGTGACCT<br>R:TCGCCGAAGAGTTGACATAG	344	MseI	37°C 1h. 2,5% gel	Khan et al., 2005a
**C22-8**	C22-8	chromosome Ib	F:TGATGCATCCATGCGTTTAT<br>R:CCTCCACTTCTTCGGTCTCA	F:TCTCTCTACGTGGACGCC<br>R:AGGTGCTTGGATATTCGC	521	BsmAI + MboII	37°C 30min, 55°C 30 min. 2,5% gel	Khan et al., 2005a
**C29-2**	C29-2	chromosome III	F:ACCCACTGAGCGAAAAGAAA<br>R:AGGGTCTCTTGCGCATACAT	F:AGTTCTGCAGAGTGTCGC<br>R:TGTCTAGGAAAGAGGCGC	446	HpyCH4IV + RsaI	37°C 1h. 2% gel	Khan et al., 2005a
**L358**	L358	chromosome o V	F:TCTCTCGACTTCGCCTCTTC<br>R:GCAATTTCCTCGAAGACAGG	F:AGGAGGCGTAGCGCAAGT<br>R:CCCTCTGGCTGCAGTGCT	418	HaeIII + NlaIII	37°C 1h. 2,5% gel	Khan et al., 2005 a,

**PK1**	PK1	chromosome VI	F:GAAAGCTGTCCACCCTGAAA<br>R:AGAAAGCTCCGTGCAGTGAT	F:CGCAAAGGGAGACAATCAGT<br>R:TCATCGCTGAATCTCATTGC	903	AvaI + RsaI	37°C 1h. 2,5% gel	Khan et al., 2005 a,
**APICO**	Apico	Apicoplast chromosome	F:ATGGTTTTAACCCTAGATTGTGG<br>R:ACGGAATTAATGAGATTTGAA	F:GCAAATTCTTGAATTCTCAGTT<br>R:GGGATTCGAACCCTTGATA	640	AflII + DdeI	37°C 1h. 2,5% gel	Dubey et al; 2007

* CutSmart Buffer® was used for all reactions; **F: forward *primer*; ***R: reverse *primer*
