# Supplementary material for: Presence of atypical genotypes of Toxoplasma gondii isolated from cats in the state of Bahia, Northeast of Brazil
Source: PLoS One. 2021 Oct 5;16(10):e0253630. doi: 10.1371/journal.pone.0253630 (PMC8491887; doi:10.1371/journal.pone.0253630)
Supplement: S2 Table — a) Based on the position within each chromosome of the ME49 strain, as indicated on the Toxo DB website (http://toxodb.org). b) (F), direct initiator; (R), reverse primer. In each primer, the direct primer was labeled in part 5 with fluoroscein: 6-carboxyfluorescein (6-FAM) for MS TUB2, XI.1, B18, N83, N61, M33 and M48, hexachlorofluorescein (HEX) for MS TgM -A, B17, N82, W35 and IV.1 and 2,7,8-benzo -5-fluoro-2, 4,7-trichloro-5-carboxyfluorescein (NED) for MS AA, N60 and M102. SOURCE: Ajzemberg et al., (2010). (PDF) [file pone.0253630.s002.pdf]

S2 Table. Microsatellite markers and PCR primers used for the PCR assay

Marker	Chromosome (position)[a]	Repeat Reason (s)	Primer Sequence [b]	Band size (bp)
TUB2	IX (974608 to 974896)	[TG/AC]$_n$	(F) 5′ 6-FAM-GTCCGGGTGTTCCTACAAAA 3′	287-291
			(R) 5′ TTGGCCAAAGACGAAGTTGT 3′	
W35	II (633241 to 633482)	[TC/AG]$_n$, [TG/AC]$_n$	(F) 5′ HEX-GGTTCACTGGATCTTCTCCAA 3′	242-248
			(R) 5′ AATGAACGTCGCTTGTTTCC 3′	
TgM-A	X (4824879 to 4825083)	[TG/AC]$_n$	(F) 5′ HEX-GGCGTCGACATGAGTTTCTC 3′	203-211
			(R) 5′ TGGGCATGTAAATGTAGAGATG 3	
B18	VIIa (2921536 to 2921693)	[TG/AC]$_n$	(F) 5′ 6-FAM-TGGTCTTCACCCTTTCATCC 3′	156-170
			(R) 5′ AGGGATAAGTTTCTTCACAACGA 3′	
B17	XII (6474746 to 6475079)	[TC/AG]$_n$	(F) 5′ HEX-AACAGACACCCGATGCCTAC 3′	334-366
			(R) 5′ GGCAACAGGAGGTAGAGGAG 3	
M33	IV (672591 to 672760)	[TC/AG]$_n$	(F) 5′ 6-FAM- TACGCTTCGCATTGTACCAG 3′	165-173
			(R) 5′ TCTTTTCTCCCCTTCGCTCT 3′	
IV.1	IV (742419 to 742693)	[TG/AC]$_n$	(F) 5′ HEX-GAAGTTCGGCCTGTTCCTC 3′	272-282
			(R) 5′ TCTGCCTGGAAAAGGAAAGA 3′	
XI.1	XI (189702 to 190058)	[TG/AC]$_n$	(F) 5′ 6-FAM-GCGTGTGACGAGTTCTGAAA 3′	354-362
			(R) 5′ AAGTCCCCTGAAAAGCCAAT 3′	
M48	Ia (332951 to 333166)	[TA/AT]$_n$	(F) 5′ 6-FAM-AACATGTCGCGTAAGATTCG 3′	209-243

			(R) 5′ CTCTTCACTGAGCGCCTTTC 3′	
M102	VIIa (3093491 to 3093664)	[TA/AT]$_n$	(F) 5′ NED-CAGTCCAGGCATACCTCACC 3′	164-196
			(R) 5′ CAATCCCAAAATCCCAAACC 3	
N60	Ib (1766079 to 1766221)	[TA/AT]$_n$	(F) 5′ NED-GAATCGTCGAGGTGCTATCC 3′	132-157
			(R) 5′ AACGGTTGACCTGTGGCGAGT 3	
N82	XII (1621472 to 1621585)	[TA/AT]$_n$	(F) 5′ HEX-TGCGTGCTTGTCAGAGTTC 3′	105-145
			(R) 5′ GCGTCCTTGACATGCACAT 3	
AA	VIII (5836880 to 5837144)	[TA/AT]$_n$	(F) 5′ NED-GATGTCCGGTCAATTTTGCT 3′	251-332
			(R) 5′ GACGGGAAGGACAGAAACAC 3′	
N61	VIIb (4217145 to 4217238)	[TA/AT]$_n$	(F) 5′ 6-FAM-ATCGGCGGTGGTTGTAGAT 3′	79-123
			(R) 5′ CCTGATGTTGATGTAAGGATGC 3′	
N83	X (1772898 to 1773209)	[TA/AT]$_n$	(F) 5′ 6-FAM-ATGGGTGAACAGCGTAGACA 3′	306-338
			(R) 5′ GCAGGACGAAGAGGATGAGA 3′	

a) Based on the position within each chromosome of the ME49 strain, as indicated on the Toxo DB website (http://toxodb.org). b) (F), direct initiator; (R), reverse primer. In each primer, the direct primer was labeled in part 5 with fluoroscein: 6-carboxyfluorescein (6-FAM) for MS TUB2, XI.1, B18, N83, N61, M33 and M48, hexachlorofluorescein (HEX) for MS TgM -A , B17, N82, W35 and IV.1 and 2,7,8-benzo -5-fluoro-2, 4,7-trichloro-5-carboxyfluorescein (NED) for MS AA, N60 and M102. SOURCE: Ajzemberg et al., (2010).
