# Supplementary material for: Presence of atypical genotypes of Toxoplasma gondii isolated from cats in the state of Bahia, Northeast of Brazil
Source: PLoS One. 2021 Oct 5;16(10):e0253630. doi: 10.1371/journal.pone.0253630 (PMC8491887; doi:10.1371/journal.pone.0253630)
Supplement: S3 Table — u-1, u-2 –atypical alleles; Nd -not determined. (PDF) [file pone.0253630.s003.pdf]

S3 Table. Different *T. gondii* genotypes using PCR-RFLP in Bahia, Brazil.

**Reference**	**Isolated**	**Molecular Marker**											
		SAG1	5’+3’SAG2	alt. SAG2	SAG3	BTUB	GRA6	c22-8	c29-2	L358	PK1	Apico	CS3
**Reference (I)**	RH	I	I	I	I	I	I	I	I	I	I	I	I
**Reference (II)**	PTG	II or III	II	II	II	II	II	II	II	II	II	II	II
**Reference (III)**	CTG	II or III	III	III	III	III	III	III	III	III	III	III	III
**Reference**	TgCgCa 1 (COUGAR)	I	II	II	III	II	II	II	u-1	I	u-2	I	II
**Reference**	MAS	u-1	I	II	III	III	III	u-1	I	I	III	I	II
**Reference**	TgCatBr 5	I	III	III	III	III	III	I	I	I	u-1	I	II
**This study**	TgCatBr 85	I	I	I	I	III	u-1	I	III	III	I	III	u-1
	TgCatBr 86	I	I	III	III	I	I	I	III	I	I	I	I
	TgCatBr 87	I	I	III	I	III	Nd	II	III	III	nd	III	I
	TgCatBr 88	I	I	III	III	III	III	II	III	I	I	III	I

	TgCatBr 89	I	I	III	u-1	I	III	II	I	u-1	u-1	I	I
**Silva et al 2017 (Dog)**	TgDgBA 6	I	III	Nd	III	I	III	I	II	I	u-2	I	Nd
	TgDgBA 13	I	III	Nd	III	II	III	I	II	III	u-2	I	Nd
	TgDgBA 17	I	II	Nd	III	II	III	u-1	II	I	u-2	I	Nd
	TgDgBA 21	I	I	Nd	III	I	III	u-1	II	III	u-2	I	Nd
**Bezerra et al (Pigs)**	TgPgBr 06, 08, 11, 12, 14, 15	I	I	Nd	III	III	Nd	I	Nd	Nd	I	III	Nd
	TgPgBr7	I	I	Nd	III	III	Nd	u-1	Nd	Nd	NA	III	Nd
	TgPgBr9	I	I	Nd	III	II	Nd	I	Nd	Nd	I	III	Nd
	TgPgBr10	u-1	I	Nd	III	III	Nd	III	Nd	Nd	I	III	Nd
	TgPgBr13	I	I	Nd	III	I	Nd	III	Nd	Nd	u-1	III	Nd
	TgPgBr16	I	I	Nd	III	I	Nd	I	Nd	Nd	I	III	Nd
**Maciel et al (Sheep)**	#54	I	I	Nd	I	III	III	III	u-1	u-1	u-1	III	Nd
	#124	I	I	Nd	I	III	III	III	u-1	u-1	u-1	III	Nd
	#127	I	I	Nd	I	III	III	III	u-1	u-1	u-1	III	Nd

**Bezerra et al 2015 (Chaetomys subspinosus)**	TgCsBr 01	I	I	Nd	III	Nd	Nd	I	Nd	Nd	III	III	Nd
	TgCsBr 01	I	I	Nd	III	Nd	Nd	III	Nd	Nd	I	III	Nd
	TgCsBr 01	I	I	Nd	III	Nd	Nd	III	Nd	Nd	I	III	Nd
**Rocha et al 2018 (Chickens)**	6 (TgCkBr 288, 289, 290, 291, 292, 293)	I	I	I	I	I	III	II	III	III	I	III	III
	2 (Tg294, 295)	I	III	III	III	I	III	u-1	I	I	I	III	I
	4 (TgCkBr 296, 301, 305, 308)	I	III	III	III	III	III	I	III	III	I	III	I
	1 (TgCkBr 302)	I	I	I	III	I	I	II	I	III	I	III	I
	1 (TgCkBr303)	I	III	III	III	III	II	I	III	III	I	III	I
	1 (TgCkBr 304)	I	I	I	III	I	III	u-1	I	I	I	I	I
	1 (TgCkBr307)	I	I	I	III	I	III	II	I	III	I	III	I
	1 (TgCkBr 300)	I	III	III	III	I	Nd	Nd	III	I	Nd	III	I
	1 (TgCkBr306)	I	III	III	III	Nd	III	Nd	Nd	Nd	I	III	I
	1 (TgCkBr284)	I	I	I	I e III	I	III	III	III	I	I	III	III
	1 (TgCkBr 287)	I	I	I	I e III	I	III	II	III	III	I	III	III
	1 (TgCkBr 297)	I	I e III	III	III	I	II	u-1	I	I	I	I	I
	2 (TgCkBr 298, 299)	I	I	II	III	III	III	I	I e III	I	II	III	I

u-1, u-2 – atypical alleles; Nd -not determined

3

4

5

6

7

8
