# Supplementary material for: Presence of atypical genotypes of Toxoplasma gondii isolated from cats in the state of Bahia, Northeast of Brazil
Source: PLoS One. 2021 Oct 5;16(10):e0253630. doi: 10.1371/journal.pone.0253630 (PMC8491887; doi:10.1371/journal.pone.0253630)
Supplement: S4 Table — Three archetypal reference strains (ENT, ME49 and NED) are included. * bold atypical alleles. (PDF) [file pone.0253630.s004.pdf]

S4 Table. Genotyping by MS of Toxoplasma gondii isolates from cats in Bahia-Brazil and compared to isolates from other studies. Three archetypal reference strains (ENT, ME49 and NED) are included.

			Microsatellite Markers														
			Typing Markers								Fingerprint Markers						
**Reference**	**Isolated**	**MS type**	**TUB2**	**W35**	**TgM-A**	**B18**	**B17**	**M33**	**IV.1**	**XI.1**	**M48**	**M102**	**N60**	**N82**	**AA**	**N61**	**N83**
	ENT	I	291	248	209	160	342	169	274	358	209	166	145	119	267	87	**308**
	ME49	II	289	242	207	158	336	169	274	356	215	174	142	111	265	91	**310**
	NED	III	289	242	205	160	336	165	278	356	215	190	147	111	267	91	**312**
**This study**																	
	TgCatBr85	Atypical	289	242	205	158	**332**	165	-	-	**211**	**162**	**131**	111	**245**	**103**	302
	TgCatBr86	Atypical	**261***	-	205	-	**332**	165	274	356	-	**164**	-	**109**	-	**101**	302
	TgCatBr87	Atypical	289	242	205	158	**332**	165	274	**352**	-	**162**	**131**	**109**	**247**	**103**	302
	TgCatBr88	Atypical	289	242	205	**144**	336	-	274	-	**211**	**164**	**131**	111	**247**	**101**	302
	TgCatBr89	Atypical	289	242	-	160	**332**	-	274	356	209	**162**	**131**	-	**245**	**103**	302
**Ajzemberg et al 2010**																	
	P	I	291	248	209	160	342	169	274	358	209	166	145	121	267	87	**308**
	CT1	I	291	248	209	160	342	169	274	358	209	168	145	119	265	87	**306**
	GIL	I	291	248	209	160	342	169	274	358	209	166	147	119	265	87	**306**
	BK	I	291	248	209	160	342	169	274	358	209	166	145	119	265	87	**316**
	NTE	II	289	242	207	158	336	169	274	356	221	176	140	113	269	103	**310**
	PTG	II	289	242	207	158	336	169	274	356	215	174	142	111	265	91	**310**
	BOU	II	289	244	207	158	336	169	274	356	213	178	140	113	259	99	**310**
	CTG	III	289	242	205	160	336	165	278	356	215	190	147	111	269	89	**312**
	NED	III	289	242	205	160	336	165	278	356	209	190	147	111	267	91	**312**
	M7741	III	289	242	205	160	336	165	278	356	215	190	147	111	267	91	**312**
	VEG	III	289	242	205	160	336	165	278	356	213	188	153	111	267	89	**312**
	MAS	Atypical	291	242	205	162	362	169	272	358	221	166	142	111	332	95	**338**
	CASTELLS	Atypical	287	242	207	158	358	169	274	356	239	164	138	109	283	87	**324**
	TgCatBr1	Atypical	289	242	205	160	342	165	278	358	233	164	147	111	316	89	**308**
	GUY-KOE	Atypical	289	246	203	160	337	165	274	356	209	172	136	111	251	109	**310**

S4 Table cont.			*Microsatellite Markers*														
			**Typing Markers**								**Fingerprint Markers**						
**Reference**	Isolated	**MS type**	**TUB2**	**W35**	**TgM-A**	**B18**	**B17**	**M33**	**IV.1**	**XI.1**	**M48**	**M102**	**N60**	**N82**	**AA**	**N61**	N83
	GUY-MAT	Atypical	291	242	203	160	339	165	272	358	221	174	138	107	277	95	312
	RUB	Atypical	289	242	205	170	360	167	274	356	223	190	142	109	259	85	312
	GPHT	Atypical	291	248	205	160	342	165	274	354	229	166	147	111	283	91	306
	BOF	Atypical	291	248	205	160	342	165	274	354	227	166	147	111	273	89	306
	CAST	Atypical	291	242	205	158	342	167	276	356	211	168	147	119	279	87	306
	TgCatBr5	Atypical	291	242	205	160	362	165	278	356	237	174	140	111	265	89	314
	P89	Atypical	291	242	205	160	348	165	278	356	213	190	142	111	261	87	314
	TgCatBr3	Atypical	289	242	205	160	348	165	278	356	213	190	142	111	263	113	312
	VAND	Atypical	291	242	203	162	344	167	276	356	217	170	142	113	277	91	308
	GUY-DOS	Atypical	289	246	203	160	344	167	272	356	229	176	142	113	263	85	312
**Can et al 2014**																	
	TgCatTr_Izmir 1	(Type II)	289	242	207	158	336	169	274	356	227	176	142	117	263	87	314
	TgCatTr_Izmir 2	(Type II)	289	242	207	158	336	169	274	356	227	172	145	131	263	99	310
	TgCatTr_Izmir 3	(Type II)	289	242	207	158	336	169	274	356	227	176	140	115	279	95	312
	TgCatTr_Izmir 4	(Africa 1)	291	248	205	160	342	-	274	354	231	166	147	111	295	91	310
	TgCatTr_Izmir 5	(Type II)	289	242	207	158	336	169	274	356	223	176	142	-	273	87	314
	TgCatTr_Izmir 6	(Type II)	289	242	207	158	336	169	274	356	211	176	140	123	275	85	314
	TgCatTr_Izmir 7	(Type II)	289	242	207	158	336	169	274	356	213	178	140	111	263	97	316
	TgCatTr_Izmir 8	(Type II)	289	242	207	158	336	169	274	356	219	176	142	123	-	85	310
	TgCatTr_Izmir 9	(Type II)	289	242	207	158	336	169	274	356	213	178	140	119	295	101	310
	TgCatTr_Izmir 10	(Type III)	289	242	205	160	336	165	278	356	213	190	147	111	267	87	314
	TgCatTr_Izmir 11	(Type II)	289	242	207	158	336	169	274	356	223	176	142	127	277	85	314
	TgCatTr_Izmir 12	(Type II)	289	242	207	158	336	169	274	356	215	178	138	109	297	109	310
	TgCatTr_Izmir 13	(Type II)	289	242	207	158	336	169	274	356	213	176	140	117	259	87	314
	TgCatTr_Izmir 14	(Type III)	289	242	205	160	336	165	278	356	213	190	149	111	265	91	312
	TgCatTr_Izmir 15	(Type II)	289	242	207	158	336	169	274	356	227	176	140	115	265	97	312
	TgCatTr_Izmir 16	(Type II)	289	242	207	158	336	169	274	356	221	176	142	115	271	103	312
	TgCatTr_Izmir 17	(Type II)	289	242	207	158	336	169	274	356	213	174	140	109	265	91	308
	TgCatTr_Izmir 18	(Type II)	289	242	207	158	336	169	274	356	215	174	140	125	261	87	314

S4 Table cont.			*Microsatellite Markers*														
			Typing Markers								Fingerprint Markers						
**Reference**	Isolated	**MS type**	**TUB2**	**W35**	**TgM-A**	**B18**	**B17**	**M33**	**IV.1**	**XI.1**	**M48**	**M102**	**N60**	**N82**	**AA**	**N61**	**N83**
	TgCatTr_Izmir 19	(Type II)	289	242	207	158	336	169	274	356	231	174	142	111	263	97	314
	TgCatTr_Izmir 20	(Type II)	289	242	207	158	336	169	274	356	241	176	140	113	271	89	310
	TgCatTr_Izmir 21	(Type II)	289	242	207	158	336	169	274	356	213	172	145	111	267	97	310
	TgCatTr_Izmir 22	(Type II)	289	242	207	158	336	169	274	356	213	172	145	111	267	97	310
**Pena et al 2017**																	
	PS-TgCatBrSC1	I variant	291	248	209	160	334	167	274	358	223	166	145	129	269	087	306
**Silva et al 2017**																	
	TgCatBrFN1		289	242	207	158	336	169	274	356	245	178	138	123	271	095	329
	TgRatnoBrFN1		289	242	207	158	336	169	274	356	235	178	138	123	271	097	329
	TgRatnoBrFN2		289	242	209	160	336	165	278	356	213	190	145	111	267	087	308
	TgRatRaBrFN1		291	242	205	162	342	165	278	356	213	164	142	109	279	087	312
**Pena et al 2020**																	
	TgMytrBrSP1	Atypical	289	242	205	160	348	165	278	356	213	190	142	111	263	103	312
**Valenzuela-Moreno et al. 2020**																	
	TgDogMxChp3		*291*	*242*	*205*	*160*	*348*	*165*	*278*	*356*	213	190	145	111	261	87	314
	TgDogMxChp11		291	248	205	158	342	165	276	358	211	168	147	119	277	89	314

* bold atypical alleles
